# Staphylococcus epidermidis bacteremia after small bowel enteroscopy

**DOI:** 10.1055/a-2420-7848

**Published:** 2024-10-14

**Authors:** Saif Ullah, Faisal Shaukat Ali, Xin-Guang Cao, Fangbin Zhang

**Affiliations:** 1 191599 Gastroenterology, The First Affiliated Hospital of Zhengzhou University, Zhengzhou, China; 2 12340 Gastroenterology, Hepatology, and Nutrition, The University of Texas Health Science Center at Houston, Houston, United States

A 61-year-old man with a 6-month history of recurrent hematochezia was admitted to our hospital for the management of symptomatic anemia. Upon admission, his hemoglobin level was 60 g/L. Esophagogastroduodenoscopy and colonoscopy were performed but did not reveal the cause of his anemia. Subsequently, single-balloon enteroscopy (SBE) was conducted, which identified an actively bleeding angioectasia in the distal jejunum, approximately 300 cm from the incisors ( Fig. 1 **a** , Video 1 ). Hemostasis was achieved using argon plasma coagulation and hemoclip placement at the bleeding site ( Fig. 1 **b, c** ). The SBE procedure time was approximately 13 min. Two days post-SBE, the patient developed a fever, peaking at 39°C, along with leukocytosis (white blood cell count: 21 × 10 ^9^ /L). A computed tomography (CT) scan of the chest and abdomen did not reveal any source of infection.

**Fig. 1:**
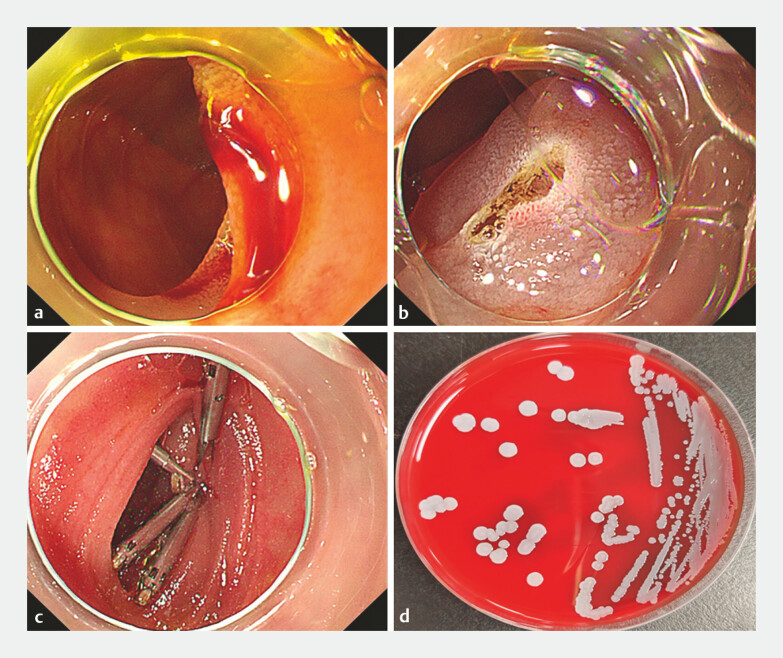
Single balloon enteroscopy (SBE) was performed to evaluate the cause of anemia in a 61-year-old ma. **a** SBE showed an active bleeding angioectasia in the distal jejunum. **b** Hemostasis was achieved using argon plasma coagulation. **c** Wound closure after hemostasis using endoclips. **d** Peripheral venous blood culture revealed growth of *Staphylococcus epidermidis* .

Single balloon enteroscopy showing an active bleeding angioectasia in the distal jejunum in a 61-year-old man with anemia.Video 1

Blood cultures grew Staphylococcus epidermidis in three consecutive samples ( Fig. 1 **d** ). The patient was treated with an initial intravenous dose of tigecycline at 100 mg, followed by 50 mg every 12 h. He became afebrile 2 days after starting antibiotics. At the 3-month follow-up, the patient remained afebrile with no recurrence of bacteremia.

SBE is commonly performed for both diagnostic and therapeutic interventions in the small intestine 1 . Despite disinfection and processing of endoscopes, device-associated infections can still occur. Notably, an overtube is used during SBE, which is placed on the endoscope in a non-sterile environment. Inflation of the balloon used to advance the enteroscope can compress the intestinal wall, and prolonged balloon inflation (> 10 min) may cause intestinal wall hypoxia, potentially disrupting the mucosal barrier and increasing the risk of bacterial translocation, which can result in bacteremia 1 2 3 . Additionally, mucosal injury is frequently encountered during SBE, further facilitating bacterial translocation 4 .

The incidence of S. epidermidis infections has risen with the increased use of medical instrumentation. Our patient had no cutaneous lesions or evidence of accompanying gastrointestinal or respiratory infections. Therefore, we attributed the S. epidermidis bacteremia to the introduction of the bacteria into the digestive tract during the SBE procedure, followed by translocation through the damaged intestinal mucosa.

Given the increased use of balloon-assisted enteroscopy, our case highlights the importance of being aware of the potential for enteroscopy-associated bacteremia. Early recognition of this complication can facilitate timely initiation and discontinuation of antimicrobial therapy.

Endoscopy_UCTN_Code_CPL_1AI_2AD

## Notes

Endoscopy E-Videos https://eref.thieme.de/e-videos
*E-Videos* is an open access online section of the journal *Endoscopy* , reporting on interesting cases and new techniques in gastroenterological endoscopy. All papers include a high-quality video and are published with a Creative Commons CC-BY license. Endoscopy E-Videos qualify for HINARI discounts and waivers and eligibility is automatically checked during the submission process. We grant 100% waivers to articles whose corresponding authors are based in Group A countries and 50% waivers to those who are based in Group B countries as classified by Research4Life (see: https://www.research4life.org/access/eligibility/ ). This section has its own submission website at https://mc.manuscriptcentral.com/e-videos .

## References

[1] Lee TC Huang YC Lu YZ Hypoxia-induced intestinal barrier changes in balloon-assisted enteroscopy J Physiol 2018 596 3411 3424 10.1113/JP275277 29178568 PMC6068115

[2] Huang CY Hsiao JK Lu YZ Anti-apoptotic PI3K/Akt signaling by sodium/glucose transporter 1 reduces epithelial barrier damage and bacterial translocation in intestinal ischemia Lab Invest 2011 91 294 309 10.1038/labinvest.2010.177 20975661

[3] Lu YZ Huang CY Huang YC Tumor necrosis factor α-dependent neutrophil priming prevents intestinal ischemia/reperfusion-induced bacterial translocation Dig Dis Sci 2017 62 1498 1510 10.1007/s10620-017-4468-3 28144894

[4] Abu Taleb AMF Mohamed MS Abdel-Latif RS The role of ica operon and biofilm formation in coagulase negative staphylococcal infection Egyptian J Med Microbiol 2012 21 21 32

